# Prevalence, treatment rates and control of dyslipidemia among diabetes patients in northern Nigeria: A cross-sectional, multicenter study

**DOI:** 10.1371/journal.pone.0346350

**Published:** 2026-08-05

**Authors:** Nura Hamidu Alkali, Andrew Enemako Uloko, Godwin O. Osaigbovo, Adamu Girei Bakari, Mohammed Raji Bello, Maria Ahuoiza Garba, Garba Musa Fika, Abubakar Sahabi Muhammad, Muhammad Arabi Saad, Vandi Ghyi Zira, Umar Faruk Abdullahi, Ayuba Mugana, Ijuptil Chiroma, Ibrahim Abdullahi Haladu, Lumsami Shadrach, Usman Adamu Nuhu, Godiya Ishaya Dare

**Affiliations:** 1 Department of Medicine, Abubakar Tafawa Balewa University Bauchi, Bauchi State, Nigeria; 2 Department of Medicine, Abubakar Tafawa Balewa University Teaching Hospital Bauchi, Bauchi State, Nigeria; 3 Department of Medicine, Bayero University Kano, Kano State, Nigeria; 4 Department of Medicine, Aminu Kano Teaching Hospital Kano, Kano State, Nigeria; 5 Department of Medicine, University of Jos, Jos, Plateau State, Nigeria; 6 Department of Medicine, Ahmadu Bello University Zaria, Kaduna State, Nigeria; 7 Department of Medicine, Modibbo Adama University Teaching Hospital, Yola, Adamawa State, Nigeria; 8 Department of Anesthesia, Modibbo Adama University Teaching Hospital Yola, Adamawa State, Nigeria; 9 Department of Pediatrics, Ahmadu Bello University Zaria, Kaduna State, Nigeria; 10 Department of Medicine, Yobe State University Damaturu, Yobe State, Nigeria; 11 Department of Medicine, Modibbo Adama University Yola, Adamawa State, Nigeria; 12 Department of Medicine, University of Maiduguri, Maiduguri, Borno State, Nigeria; Johns Hopkins: Johns Hopkins University, UNITED STATES OF AMERICA

## Abstract

**Objectives:**

Dyslipidemia is prevalent among Nigerians with diabetes mellitus (DM), but its treatment has not been well-studied. The objective of this study was to determine the prevalence, treatment rates and control of dyslipidemia among DM patients in northern Nigeria.

**Methods:**

We conducted a multicenter, cross-sectional study of dyslipidemia in DM patients, and noted cardiovascular disease (CVD) risk factors, lipid-lowering treatments, body mass index, blood pressure, HbA1c, lipid profile, glomerular filtration rate and proteinuria. Outcome measures were the rate and treatment of dyslipidemia and attainment of low density lipoprotein cholesterol target for primary prevention of CVD. Binomial logistic regression was used to analyze associations between participant characteristics and dyslipidemia. Hosmer-Lemeshow goodness-of-fit test was used to evaluate the model fit. Statistical analysis was performed with the SPSS version 25 program.

**Results:**

The study enrolled 403 participants (58.8% females), of whom 59.6% had dyslipidemia. Besides DM and dyslipidemia, other risk factors for CVD were hypertension (56.8%), obesity (52.6%), chronic kidney disease (36.5%), atrial fibrillation (7.9%), heart failure (5.0%), cigarette smoking (4.7%), excess alcohol use (2.0%), and previous CVD (14.4%). Logistic regression analysis showed dyslipidemia was significantly associated with female gender (odds ratio = 1.68, *P* = 0.029) and proteinuria (odds ratio = 2.26, *P* = 0.004). Among those with dyslipidemia, 51.3% took lipid-lowering treatments comprising statins (50.8%) and clofibrate (2.9%). None took other lipid-lowering treatments, and only 17.1% attained the target for primary prevention of CVD.

**Conclusion:**

Three-fifths of patients had dyslipidemia, but only a sixth attained the treatment target. Treatment for dyslipidemia included statins and fibrates, but not niacin, ezetimibe, bempedoic acid, icosapent ethyl, inclisiran or PCSK9 inhibitors recommended for those who failed intensive statin therapy. There is the need for better access to non-statin treatment and physician adherence to clinical practice guidelines.

## Introduction

Diabetes mellitus (DM) is a chronic metabolic disease and a major cause of death and disability worldwide [1]. Up to 6.1% of the global adult population had DM in 2021, which was expected to rise to 10% or more in developing countries by 2050 [2]. DM prevalence in children is also rising worldwide. The Global Burden of Disease study has found that the prevalence of childhood DM rose by 39.4% between 1990 and 2019, with the highest rise seen in low-income countries [3].

DM is associated with high risks of cardiovascular diseases (CVD), including stroke, myocardial infarction, peripheral arterial disease (PAD), heart failure and pulmonary thrombo-embolism (PTE) [1]. Indeed, DM contributed 20% to the population attributable risk of stroke in Nigeria, while a UK study found CVD events and mortality were two to three-fold higher among DM patients than the general population [4,5]. In other studies, DM patients with no history of CVD had a similar risk of myocardial infarction as non-DM subjects with a history of CVD, suggesting that CVD risk due to DM is equivalent to a previous CVD [6]. The high CVD risks associated with DM result from premature atherosclerosis complicating the chronic effects of hyperglycemia, dyslipidemia, hypertension, obesity, and other metabolic derangements [7]. For instance, in the Action for Health in Diabetes (Look AHEAD) study, DM patients with dyslipidemia had 1.3 and 1.5 times additional risks of stroke and coronary heart disease (CHD) compared to those with DM alone [8]. Other effects of dyslipidemia in people with DM are retinopathy, macular edema, erectile dysfunction, diabetic foot syndrome and fetal anomalies during pregnancy [9–11].

Dyslipidemia is defined as high levels of serum low density lipoprotein cholesterol (LDLC), total cholesterol (TC) and/ or triglycerides (TG), or low levels of high density lipoprotein cholesterol (HDLC) [12]. The major determinants of lipoprotein levels are dietary intake, obesity, insulin resistance and genetic factors influencing lipid absorption, transport and metabolism [12]. Among genetic factors causing high LDLC are defects of the enzyme lipoprotein lipase that degrades LDL-rich TGs and very low density lipoproteins (VLDL) within chylomicrons [13,14]. Regardless of cause, excess LDLC activates macrophages to release pro-inflammatory cytokines, including vascular cell adhesion molecule-1 and interleukin-1, that further stimulate macrophages to engulf LDLC, transform into foam cells and form atherogenic plaques in the tunica media [7,13]. Platelet aggregation at the sites of atherogenic plaques subsequently leads to vascular thrombosis. Raised serum levels of apolipoproteins A and B also promote thrombogenesis. While apolipoprotein A promotes atherogenesis, apolipoprotein B competes with plasminogen and tissue plasminogen activator for binding to fibrin, thereby inhibiting plasmin-mediated fibrinolysis [14,15].

The prevalence of DM-related dyslipidemia ranges from 43.5% among type 1 DM patients in Spain to 95% among type 2 DM patients in Jordan [16,17]. Prevalence rates in Nigeria are as high as 72.6%, although the average rate across sub-Saharan Africa is 52.7% [18,19]. Thus, cardiovascular risk reduction in DM patients with dyslipidemia is a major goal of DM care, with current guidelines recommending statins as first-line treatment [18–20]. The Cholesterol Treatment Trialists’ Study has shown that use of statin to lower raised LDLC caused proportionate reductions in coronary events and stroke, while the ODYSSEY trial showed that add-on therapy with a PCSK9 inhibitor caused fewer ischemic stroke, transient ischemic attack (TIA), CHD, and fatal myocardial infarction [20–22]. Other studies support the use of niacin, fibrates, ezetimibe and bempedoic acid as alternate or add-on therapies [23–25]. Yet, evidence of this practice is scarce in Nigeria, where studies have assessed the prevalence of dyslipidemia in DM patients, but not the rates or outcomes of statin therapy [19,26–29]. A study from Nigeria also found that only 19% of stroke patients with dyslipidemia had taken LLTs, suggesting poor care of dyslipidemia in the non-DM population as well [30].

In this study, we aimed to determine the prevalence and treatment rates of dyslipidemia among DM patients in northern Nigeria, and the proportion of patients that attained the LDLC goal and target recommended for the primary prevention of CVD. Findings from this study could aid the care of dyslipidemia among Nigerians living with DM.

## Materials and methods

### Study design and setting

We conducted a cross-sectional, observational study of CVD risks and metabolic outcomes among patients at the DM clinics of Abubakar Tafawa Balewa University Teaching Hospital Bauchi (ATBUTH), Ahmadu Bello University Teaching Hospital Zaria (ABUTH), Aminu Kano Teaching Hospital Kano (AKTH), Modibbo Adama University Teaching Hospital Yola (MAUTH), and the University of Maiduguri Teaching Hospital (UMTH), Maiduguri, Nigeria. The study was conducted from 8^th^ February to 15^th^ December, 2023. Findings on CVD risks and exercise were published previously [31].

The present study utilized data on the prevalence and treatment of dyslipidemia, and the proportion of patients that attained the LDLC goal and target recommended for the primary prevention of CVD during routine care of DM. The study sites were in Bauchi, Kaduna, Kano, Adamawa and Borno states of Nigeria, a country of 923,768 square kilometers comprising 36 states and a Federal Capital Territory. The DM clinics operated once or twice weekly, and were all staffed by physician diabetologists, physiotherapists, nutritionists, DM care nurses and counsellors. Each clinic had point-of-care testing for glycated hemoglobin (HbA1c), fasting blood glucose (FBG) and urinalysis.

### Study population and sampling

Participants were recruited consecutively after signing a written, informed consent. Parents or guardians signed consent and filled study questionnaires for those aged below 18 years. The sample size was calculated using Cochran’s formula for unknown proportions: *n* = Z^2^ x p (1-p)/e^2^, where *n* is the minimum required sample size, *Z* is the standard normal (1.96) corresponding to a 95% confidence interval (CI), *p* is the unknown proportion of DM patients with dyslipidemia in the study area (0.5), and *e* is the margin of error, placed at 5% [32]. Thus, required sample size was *n* = (1.96)^2^ x 0.5(1–0.5)/0.05^2^ = 385. We anticipated a 4% attrition rate and added 16 participants, which raised the final sample size to 401.

### Inclusion and exclusion criteria

Patients were included in the study if they were registered at the DM Clinic, had consented to participate and attended two or more follow-up visits during the study. Those who missed two follow-ups or did not perform lipid profile tests were excluded from the study.

### Study instruments and variables

Data were collected on a standard questionnaire, which noted participants’ socio-demography, past medical history, duration of DM diagnosis, type of DM, complications of DM, history of dyslipidemia, hypertension, chronic kidney disease (CKD), angina, myocardial infarction (MI), stroke, PAD, PTE, illicit drug use, cigarette smoking and use of alcohol (S1 Fig.). Other items were the treatment history, including the use of insulin, non-insulin injectables, oral glucose-lowering drugs (GLD), antihypertensive drugs and LLTs. Examination and laboratory findings included weight and height measurements, body mass index (BMI), waist circumference, blood pressure (BP), serum FBG, HbA1c, TC, LDLC, HDLC, TG, electrolytes, urea, creatinine and urinalysis. The glomerular filtration rate (GFR) was calculated from participant’s creatinine values, age and sex using an online eGFR calculator of the U.S. National Kidney Foundation (https://www.kidney.org/ professionals/ gfr_calculator). An eGFR ≤ 59 ml/minute/1.73 M^2^ body surface area, constituted CKD and a risk factor for CVD [15].

### Data collection procedures

Weight and height were measured with the participant standing on a stadiometer, wearing light clothing and no shoes. The BMI was calculated from the weight in kilograms divided by the square of the height in meters. Waist circumference was taken with a non-stretching tailor’s tape placed midway between the lower rib margins and the anterior superior iliac spines. Obesity was defined as BMI of 30 kg/M^2^ or more, or waist circumference reaching 102 cm in males or 88 cm in females. The BP was the average of three readings taken with a mercury sphygmomanometer applied to the arm. Blood and urine specimens were sampled at the DM clinic after an overnight fast and tested for FBG, HbA1c and urinalysis, or sent to laboratories for tests on lipid profile, electrolytes, urea and creatinine.

### Operational terms and definitions

Type 1 and type 2 DM were defined according to the methods of Rawshani et al [33]. Type 1 DM was treatment with insulin and DM diagnosis at age < 30 years, and type 2 DM was treatment with diet, with or without oral GLD, or treatment with insulin with or without oral GLD in patients who were 40 years or older at DM diagnosis. HbA1c ≤ 7.0% was defined as good glycemic control, and higher values as poor control. Excess use of alcohol was defined as alcohol intake > 56 grams daily or 196 grams weekly in men, or 26 grams daily/ 98 grams weekly in women [34]. Dyslipidemia was defined as serum TC > 6.2 mmol/L, LDLC > 4.1 mmol/L, TG ≥ 2.3 mmol/L or HDLC < 1.0 mmol/L in males or 1.3 mmol/L in females [35]. Normal lipoprotein values excluded dyslipidemia regardless of history.

### Outcome measures

The main outcome measures were: 1. The rates and patterns of dyslipidemia, 2. The proportion of patients treated for dyslipidemia, and 3. The proportion of patients, who at enrolment, had attained the LDLC goal and target of < 1.8 mmol/L for primary prevention of CVD in people with DM as recommended by the European Society of Cardiology/ European Atherosclerosis Society [15].

### Statistical analysis

Data were analyzed with the Statistical Package for the Social Sciences (SPSS) version 25 program (IBM Corporation, New York, USA). Categorical variables were analyzed with the Pearson chi-square test or Fisher’s exact test, while means of continuous variables were analyzed with the Student’s *t* test. Binomial logistic regression was used to analyze associations between outcome measures and potential participant characteristics with P values less than 0.10 in chi square tests as well as those implicated in other studies. Calibration was assessed using the Hosmer-Lemeshow goodness-of-fit test, where a high *P* value (>0.05) was interpreted as a good fit for the model. *P* values less than 0.05 were considered significant. A four-set Venn diagram was used to display intersections of low HDLC, high TC, high LDLC and high TG counts defining dyslipidemia [36]. Results were presented as proportions, odd ratios (OR), means, medians, standard deviations (SD) and 95% confidence intervals (CIs) in tables, figures and text. The study has followed the protocol of the STROBE guidelines (S2 Fig) [37].

### Ethical considerations

The study was approved by the Health Research Ethics Committee of ATBUTH (Ref. 005/2023, dated 11^th^ January, 2023), ABUTH (Ref. ABUTHZ/HREC/F41/2023, dated 12^th^ January, 2023), UMTH (Ref. OHRP-IRB00013572 UMTH/REC/23/1110, dated 31^st^ January, 2023), MAUTH (Ref. HREC/23/239, dated 3^rd^ February, 2023), and AKTH (Ref. NHREC/28/01/2020/AKTH/EC/3532, dated 14^th^ March, 2023). The study protocol conformed to the Helsinki Declaration and PLOS human participants research checklist (S3 Fig).

## Results

### Demographic and clinical characteristics

**Fig 1:**
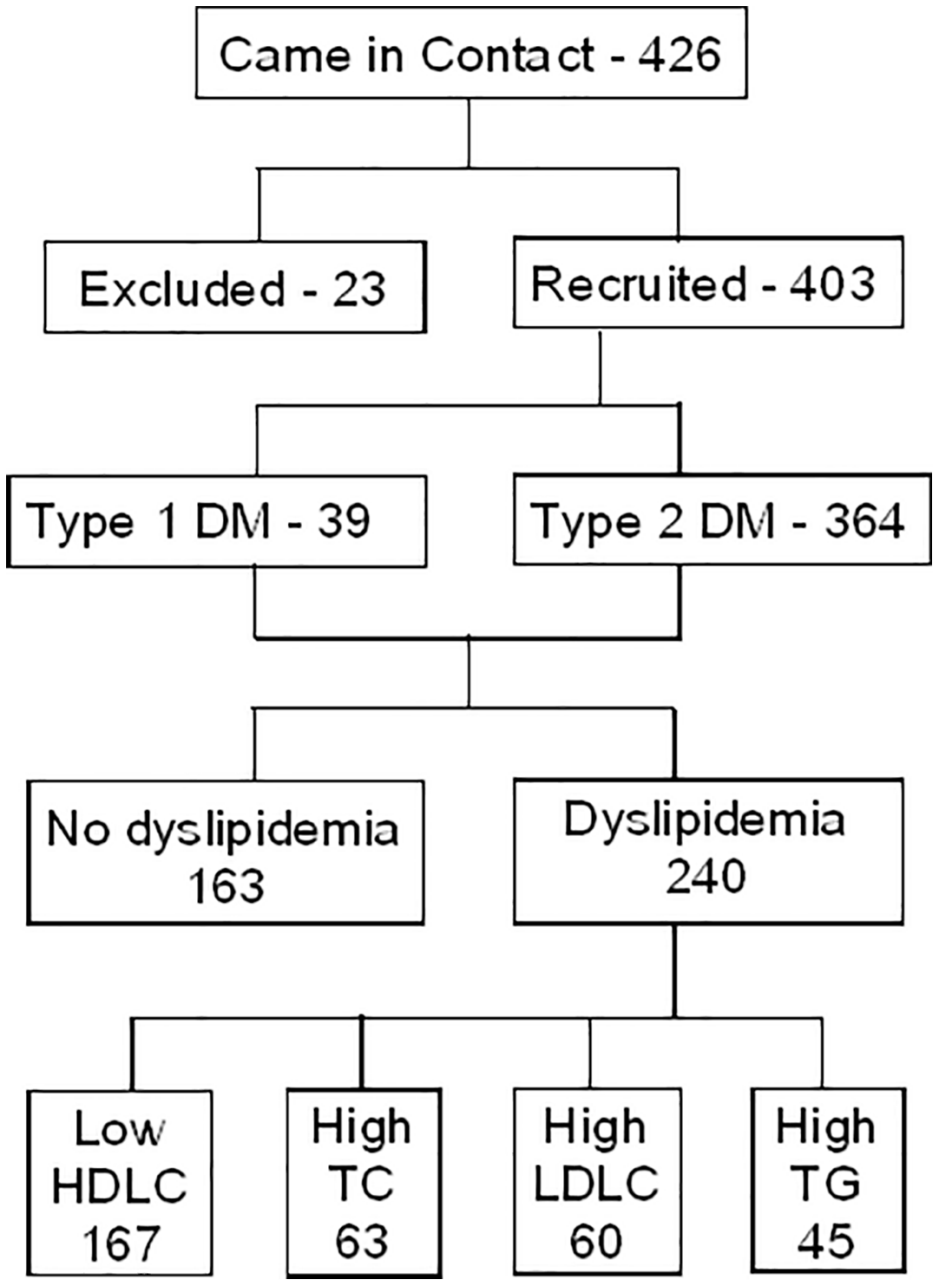
Flowchart of study participants. DM, Diabetes mellitus; HDLC, High density lipoprotein cholesterol; LDLC, Low density lipoprotein cholesterol; TC, Total cholesterol; TG, Triglycerides.

We enrolled 403 patients (58.8% females) out of 426 screened for the study. Thus, the response rate was 94.6% (Fig 1).

Mean age of participants (±SD) was 53.2 ± 12.7 years, with males older than females (54.9 ± 11.7 years vs. 52.1 ± 13.3 years, respectively; 95% CI: 0.35–5.39, *P* = 0.026) and two-thirds being urban residents. Other demographic characteristics are shown on Table 1. Those excluded from the study were similar to participants in age (mean age ± SD, 47.8 ± 16.7 vs. 53.2 ± 12.7 years, respectively; 95% CI: –0.05–10.89; *P* = 0.052), gender ratio (females, 56.5% vs. 58.8%, respectively; χ^2^ = 0.047, *P* = 0.83) and place of residence (urban area, 78.3% vs. 70.0%, respectively; χ^2^ = 0.72, *P* = 0.49). Beside DM and dyslipidemia, CVD risk factors included hypertension (56.8%), obesity (52.6%), CKD (36.5%), atrial fibrillation (7.9%), heart failure (5.0%), cigarette smoking (4.7%), excess alcohol use (2.0%), and a past history of CVD (14.4%). Other clinical and laboratory characteristics of participants are shown on Table 2.

**Table 1:** Comparison of participant demographic characteristics stratified by gender.

Characteristic, *n* (%)	Total (N = 403)	Male (*n* = 166)	Female (*n* = 237)	*χ*^2^ Statistic	*P*-Value
Study site, n (%)					
Bauchi	64 (15.9)	30 (18.1)	34 (14.3)	3.06	0.55
Kano	117 (29.0)	42 (25.3)	75 (31.6)		
Maiduguri	77 (19.1)	35 (21.1)	42 (17.7)		
Yola	75 (18.6)	29 (17.5)	46 (19.4)		
Zaria	70 (17.4)	30 (18.1)	40 (16.9)		
Place of residence, n (%)					
Urban area	282 (70.0)	110 (66.3)	172 (72.6)	1.85	0.186
Rural area	121 (30.0)	56 (33.7)	65 (27.4)		
Educational qualification, n (%)					
Primary school certificate	45 (11.2)	8 (4.8)	37 (15.6)	30.63	< 0.001*
Secondary school certificate	93 (23.1)	43 (25.9)	50 (21.1)		
University degree/diploma	154 (38.2)	76 (45.8)	78 (32.9)		
Postgraduate qualification	37 (9.2)	22 (13.25)	15 (6.3)		
No formal education	74 (18.4)	17 (10.2)	57 (24.1)		
Occupation, n (%)					
Student/ Youth Service Corps	13 (3.2)	2 (1.2)	11 (4.6)	36.94	< 0.001*
Farmer/ trader/ artisan	64 (15.9)	26 (15.7)	38 (16.0)		
Civil servant/ public servant	111 (27.5)	55 (33.1)	56 (23.6)		
Private company employee	12 (3.0)	3 (1.8)	9 (3.8)		
Police, military, paramilitary	13 (3.2)	12 (7.2)	1 (0.4)		
Business person/contractor	56 (13.9)	31 (18.7)	25 (10.5)		
Retiree/unemployed/FTHW	134 (33.25)	37 (22.3)	97 (40.9)		
Marital status					
Single	22 (5.5)	8 (4.8)	13 (5.5)	33.05	< 0.001*
Married	320 (79.4)	153 (92.2)	168 (70.9)		
Divorced/Widowed	61 (15.1)	5 (3.0)	56 (23.6)		

* Significant p-value; FTHW, Full-time housewife

**Table 2:** Cardiovascular risk factors among participants compared by χ^2^ and Student *t* tests.

A. Categorical variables, n (%)	Total (N = 403)	Male (*n* = 166)	Female (*n* = 237)	*χ*^2^ Statistic	*P-* Value
Type of DM					
Type 1	39 (9.7)	11 (6.6)	28 (11.8)	3.0	0.09
Type 2	364 (90.3)	155 (93.4)	209 (88.2)		
Old age (≥ 65 years)	79 (19.6)	32 (19.3)	47 (19.8)	0.02	1.0
Obesity	209/397 (52.6)	48/162 (29.6)	161/235 (68.5)	14.85	< 0.001*
Dyslipidemia	240 (59.6)	84 (50.6)	156 (65.8)	9.389	0.003*
Hypertension	229 (56.8)	89 (53.6)	140 (59.1)	1.18	0.307
Atrial fibrillation	32 (7.9)	15 (9.0)	17 (7.2)	0.464	0.575
Heart failure	20 (5.0)	9 (5.4)	11 (4.6)	0.126	0.817
Cigarette smoking	19 (4.7)	19 (11.4)	0 (0.0)	28.5	< 0.001*
Excess use of alcohol†	8 (2.0)	7 (4.2)	1 (0.4)	7.23	0.01*
Past history of CVD	58 (14.4)	22 (13.2)	36 (15.2)	0.3	0.67
Chronic kidney disease	142/389 (36.5)	52/161 (32.3)	90/228 (39.5)	2.1	0.165
Proteinuria	160/402 (34.8)	67 (40.4)	93/236 (39.4)	0.037	0.918
**B. Continuous variables,** **Mean (SD)**	**Total** **(N = 403)**	**Male** **(*n* = 166)**	**Female** **(*n* = 237)**	**95% CI**	***P* Value**
Age, years	53.2 (12.7)	54.9 (11.7)	52.1 (13.3)	0.35–5.39	0.026*
Duration of DM, months	100.4 (85.3)	104.0 (88.2)	97.8 (83.4)	− 4.8–23.1	0.48
Body mass index, kg/M^2^	28.5 (5.8)	26.4 (4.8)	29.9 (6.1)	2.3–4.7	< 0.001*
WC, centimeter	*N/A*	94.5 (13.1)	95.7 (15.4)	*N/A*	*N/A*
Systolic BP, mm/Hg	131.7 (18.9)	132.6 (18.5)	131.2 (19.2)	−2.82–5.71	0.51
FBG, mmol/L	8.8 (4.1)	8.5 (4.0)	9.0 (4.1)	−1.31–0.3	0.22
HbA1c, %	8.7 (2.1)	8.6 (2.1)	8.8 (2.2)	−0.69–0.2	0.27
Serum creatinine, µmol/L	117.3 (81.3)	128.7 (96.5)	109.2 (68.4)	3.1–36.0	0.02*
GFR, ml/minute/1.73M^2^	72.2 (30.4)	73.4 (29.1)	71.4 (31.3)	−4.14–8.16	0.52
Serum lipid profile, mmol/L					
Total cholesterol	5.0 (1.4)	4.9 (1.4)	5.1 (1.3)	−0.42–0.13	0.3
LDL cholesterol	2.9 (1.1)	2.8 (1.1)	2.9 (1.1)	−0.32–1.31	0.41
HDL cholesterol	1.3 (0.56)	1.28 (0.58)	1.37 (0.54)	0.16–0.21	0.09
Triglycerides	1.5 (0.88)	1.52 (0.89)	1.50 (0.88)	−0.16–0.19	0.88

*Significant p-value; †As defined in methodology and Reference [34]; BP, Blood pressure; CI, Confidence interval; DM, Diabetes mellitus. FBG, Fasting blood glucose; GFR, Glomerular filtration rate; HbA1c, Glycated hemoglobin; HDL, High density lipoprotein; LDL, Low density lipoprotein; N/A, Not applicable; SD, Standard deviation; WC, Waist circumference

### Types of diabetes, duration and treatment

Thirty-Nine (9.7%) participants had type 1 DM and 364 (90.3%) had type 2 DM. Mean age (±SD) at DM diagnosis was 24.2 ± 6.1 years for type 1 DM and 47.11 ± 9.4 years for type 2 DM (95% CI: 19.8–25.9, *P* < 0.001), while median DM duration was 77 and 84 months, respectively. DM care at enrolment involved dietary control alone (1.7%), dietary control with oral GLD only (61.8%), dietary control with insulin only (9.2%), and dietary control combined with oral GLD and either insulin (26.8%) or semaglutide (0.24%).

### Outcome measures

1. Rates and patterns of dyslipidemia

**Fig 2:**
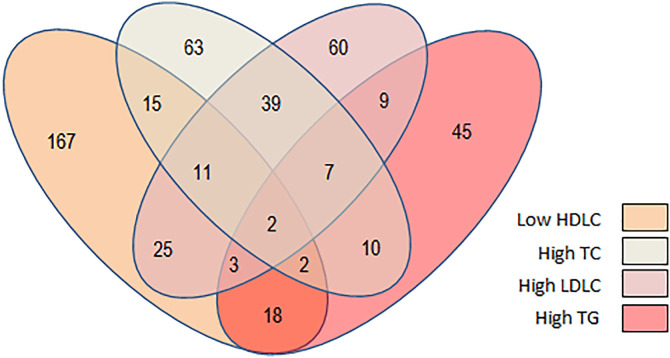
Four-set Venn diagram for lipoprotein levels of dyslipidemia in 240 participants. HDLC, High density lipoprotein cholesterol; LDLC, Low density lipoprotein cholesterol; TC, Total cholesterol; TG, Triglycerides.

Dyslipidemia was present in 240 (59.6%) participants, comprising 84 males and 156 females, with a mean age (±SD) of 53.5 ± 12.8 years (Table 2). Males were significantly older than females (55.8 ± 11.5 years vs. 52.2 ± 13.3 years, respectively; 95% CI: 0.23–7.0, *P* = 0.036). Lipoprotein levels of dyslipidemia showed a low HDLC in 69.6%, a high TC in 26.2%, a high LDLC in 25.0%, and a high TG in 18.7% participants (Fig 2).

Using chi square tests, we found that participant characteristics associated with dyslipidemia were female gender (65% females vs. 35% males; χ^2^ = 9.39, *P* = 0.003), obesity (58.6% vs. 43.7% respectively; χ^2^ = 8.51, *P* = 0.004) and proteinuria (44.8% vs. 32.5% respectively; χ^2^ = 6.074, P < 0.017), but not place of residence, age > 50 years, DM duration > 10 years, hypertension, CKD, poor glycemic control, excess alcohol use or other variables (Table 3).

**Table 3:** Chi square tests of associations between participant variables and dyslipidemia.

Variable, n (%)	Dyslipidemia (*n* = 240)	No dyslipidemia (*n* = 163)	χ^2^ Statistic	P-Value
Gender: Male	84 (35.0)	82 (50.3)	9.39	0.003*
Female	156 (65.0)	81 (49.7)		
Age > 50 years	154 (64.2)	99 (60.7)	0.49	0.53
Residence: Urban	169 (70.4)	113 (69.3)	0.05	0.83
Rural	71 (29.6)	50 (30.7)		
Tertiary education	105 (43.7)	87 (53.4)	3.61	0.067
Obesity	139/237 (58.6)	70/160 (43.7)	8.51	0.004*
Hypertension	140 (58.3)	89 (54.6)	0.551	0.261
Atrial fibrillation	20 (8.3)	12 (7.4)	0.125	0.852
Heart failure	15 (6.25)	5 (3.1)	2.085	0.168
Cigarette smoking	14 (5.8)	5 (3.1)	1.653	0.237
Excess use of alcohol^‡^	6 (2.5)	2 (1.23)	0.81	0.482
HbA1c > 7.0%	200 (83.3)	131 (80.4)	0.582	0.508
Chronic kidney disease	86/230 (37.4)	56/159 (35.2)	0.191	0.67
Proteinuria	107/239 (44.8)	53 (32.5)	6.074	0.017*
Current use of LLT	123 (56.7)	76 (33.6)	0.83	0.417
DM duration ≥ 10 years	101 (42.1)	72 (44.2)	0.173	0.683
Type of DM: Type 1	26 (10.8)	13 (8.0)	0.91	0.393
Type 2	214 (89.2)	150 (92.0)		

*Significant p-value; ‡ As defined in methodology and Reference [35]; Chronic kidney disease indicates glomerular filtration rate ≤ 59µmol/L; DM, Diabetes mellitus; HbA1c, Glycated hemoglobin; LLT, Lipid-lowering treatment

Although dyslipidemia was less common in those with university education, the difference was not statistically significant (43.7% vs. 53.4%; χ^2^ = 3.61, *P* = 0.067). Using binomial logistic regression, we analyzed associations between dyslipidemia as the dependent variable, and six participant characteristics as the independent variables, which included those with *P* values less than 0.10 in univariate analysis (female gender, university education, obesity and proteinuria) and those implicated in other studies (CKD, DM duration > 10 years). This model showed no outliers or co-linearity, and calibration with the Hosmer-Lemeshow goodness-of-fit test showed a good fit with a Chi-square statistic of 2.488 and *P* = 0.962. Variables significantly associated with dyslipidemia after controlling for the effects of confounders were female gender (*P* = 0.029) and proteinuria (*P* = 0.004), with odds ratios of 1.68 and 2.26, respectively.

2. Proportion of patients treated for dyslipidemia

At enrolment, 202 participants (50.1%) were taking LLTs, of whom 123 had dyslipidemia. Thus, use of LLT in those with dyslipidemia was 123/240 (51.3%), and included statins only (49.6%), clofibrate only (1.7%), and a statin combined with clofibrate (1.2%). The 76 participants without dyslipidemia took only statins, and none with dyslipidemia took other LLTs. Chi-square tests showed no differences among participants with dyslipidemia who did, and did not take LLTs (51.2% vs. 45.6%, respectively; χ^2^ = 0.83, *P* = 0.417).

3. Proportion of patients that attained LDLC goal and target

Among the 403 participants, 69 (17.1%) attained the LDLC goal and target for primary prevention of CVD in people with DM. Chi-square tests of association showed no significant differences in LDLC target with respect to gender (18.9% males vs. 15.2% females; χ^2^ = 1.513, *P* = 0.23), college education (16.7% vs. 17.5%; χ^2^ = 0.053, *P* = 0.89), glycemic control (15.3% good vs. 17.5% poor; χ^2^ = 0.21, *P* = 0.73), use of LLT (18.1% vs. 16.2%; χ^2^ = 0.26, *P* = 0.69) or other participant variables.

## Discussion

The study found a mean participant age of 53.2 years, with females being younger and more obese than males. The prevalence of dyslipidemia was 59.6%, and was significantly higher in females than males, in the obese, and in those with proteinuria. This prevalence was comparable to earlier findings in Nigeria, but higher than the 43.5% rate in Spain, and lower than the 62.3% rate in China [16,27,38].

Consistent with our findings of a higher rate of obesity in females, the U.S. National Health and Nutrition Examination Survey (NHANES) also found women to be more obese than men, at 40% and 35% rates, respectively [39]. Obesity being a cause of dyslipidemia, the higher rate of dyslipidemia in our female participants could be due to higher rates of obesity. Dyslipidemia was also associated with proteinuria, but not with hypertension, CKD, poor glycemic control, DM duration more than 10 years, lack of university education, cigarette smoking, excess use of alcohol or other characteristics. Previous studies have associated dyslipidemia with diabetic kidney disease and CVD [40,41]. In the study by Hirano et al, diabetic kidney disease correlated with higher risks of CVD and higher levels of serum lipoproteins, while proteinuria by itself was associated with even higher risks of these outcomes [40]. Our different results could be due to a different methodology from the Japanese study. Whereas we only used GFR to assess renal function, Hirano et al. used both GFR and the urinary albumin-to-creatinine ratio that is reportedly more sensitive to kidney injury. Meanwhile, Nigeria has the fourth highest rate of CKD worldwide, after Iran, Panama and Malaysia [42]. With hypertension, post-streptococcal glomerulonephritis and tuberculosis contributing much to the CKD burden, some of our CKD patients may have lacked diabetic kidney disease [43].

The most common form of dyslipidemia in this study was a low HDLC, and the least common was a raised TG. Previous studies from Nigeria have yielded mixed results. While some studies from the southern regions have reported low HDLC to be most common, others from the northern regions have found high LDLC and high TG to be more common [19,27,28]. Dietary practices and genetic factors influencing lipid transport and metabolism among various ethnic groups could explain these disparities. For instance, regular consumption of palm oil, a common component of the southern diet, is associated with higher levels of LDLC compared to vegetable oils low in saturated fat that are widely consumed in the north [44]. On the other hand, regular consumption of cheese in the northern regions is known to lower LDLC but also HDLC when compared to regular consumption of butter [45]. Other biologic markers that predict CVD risks associated with dyslipidemia include a high LDLC/HDLC ratio with raised levels of TGs (atherogenic dyslipidemia), and the atherogenic index of plasma derived from the logarithm of the TG/HDLC ratio [46]. The atherogenic index is reportedly a better indicator, with an index below 0.11 indicating a low risk of CVD, and higher indices indicating higher risks [46].

We observed that 51.3% participants with dyslipidemia were taking statins and other LLTs at enrolment, which was similar to the 53.8% rate in Arsi, Ethiopia, but lower than the 68% rate in Northwest China [26,47]. In contrast, studies from Turkey and Mexico found 42.4% and 23.3% treatment rates, respectively [48,49]. To our knowledge, no previous study from Nigeria has comprehensively assessed the treatment rates of dyslipidemia in a strictly DM population. However, a multinational study on lipid management in adults at risk of CHD (the INTERASPIRE study), had included Nigeria, where 40% participants had DM, and 68.6% took LLTs [25]. In that study, LLT use in Nigeria involved statins (67.3%), fibrates (0.4%), and a statin-ezetimibe combination (1.3%). We found a lower treatment rate, but the LLTs were similar, and neither study recorded the use of niacin, bempedoic acid, inclisiran, or PCSK9 inhibitors in those who failed statin therapy [15]. It remains unclear why non-statin LLTs are rarely used to treat dyslipidemia among Nigerians with DM.

We found no association between LLT use and dyslipidemia, which was expected. Beside their use in dyslipidemia, statins are also used in those lacking dyslipidemia, either as secondary prevention of CVD or as primary prevention for those at very high risks [15]. Moreover, our study protocol restricted dyslipidemia only to participants with abnormal serum lipids, regardless of current use of LLT for a previous diagnosis of dyslipidemia. Although this has excluded participants with a presumptive but false diagnosis of dyslipidemia, it did not exclude wrong use of LLT, which could mask any difference in LLT use among those with, and those without, dyslipidemia.

Finally, we found only 17.1% participants had attained the LDLC target recommended for the primary prevention of CVD in people with DM [15]. Although 14.4% of all participants needed secondary prevention due to previous CVD, those with dyslipidemia numbering only 37 were too few for a separate statistical analysis, and were jointly analyzed for primary prevention. Contrary to our findings, a Chinese study has reported a higher rate of 43.1% DM patients meeting the LDLC treatment goal and target. However, Li et al. studied only type 2 DM patients, while we studied both type 1 and type 2 DM patients [45]. Li et al. also found that lower levels of HbA1c and current use of LLT each correlated with attainment of LDLC target, which we did not find in this study, probably due to different study designs.

### Limitations of the study

Some participants had poor recall of a previous PTE, which under-estimated the rate of previous CVD. However, this didn’t impact on the main outcome measures. Secondly, we narrowly defined dyslipidemia as abnormal levels of serum lipoproteins tested during the study period, regardless of current treatment for dyslipidemia. While this excluded participant self-diagnosis of dyslipidemia on false assumptions, it did not limit wrong use of LLT. We mitigated that by assessing LLT use prescribed by physicians only. Thirdly, a few participants had missing data on BMI and creatinine, which we mitigated by excluding them during statistical analysis. Lastly, a community study would be more representative of the general population, but we lacked the financial resources for such a study.

### Summary and conclusions

Dyslipidemia was prevalent among 59.6% participants, and was significantly associated with female gender and proteinuria. Only half of those with dyslipidemia took LLTs, which comprised of statins and clofibrate only. Only 17.1% participants attained the LDLC target for primary prevention of CVD. There is a need for improved patient access to non-statin treatment and physician adherence to practice guidelines. Further studies may reveal other barriers to treating dyslipidemia at routine DM care in northern Nigeria.

## Supporting information

S1 Fig Study questionnaire. (PDF)

S2 Fig STROBE checklist. (PDF)

S3 Fig PLOS human participants research checklist. (PDF)
