# Supplementary material for: Prevalence, treatment rates and control of dyslipidemia among diabetes patients in northern Nigeria: A cross-sectional, multicenter study
Source: PLoS One. 2026 Aug 5;21(8):e0346350. doi: 10.1371/journal.pone.0346350 (PMC13440874; doi:10.1371/journal.pone.0346350)
Supplement: Supplementary file 1 [file pone.0346350.s001.pdf]

CARDIOVASCULAR RISKS IN DIABETES STUDY IN NORTHERN NIGERIA – ATBUTH BAUCHI

A. BIODATA:

Name [*Initials Only*]:	Age:	Sex:	Hosp. No:	Phone:
Residence (Town/Village):			L.G.A:	State:
Occupation: [✓] Tick one:	Student	Trader/Farmer/ Artisan	Civil Servant/Public Official	Private Organization (Private School, Bank, Company, etc)
	Military/Paramilitary		Business person	Unemployed/Retired
Educational Qualification: [✓] Tick one:	Primary/Secondary School Certificate			Tertiary education (Diploma/Degree)
	Postgraduate degree (PGD, Masters, M.Phil., Ph.D.)			No formal education
Marital Status:	Single [ ]		Married [ ]	Divorced/Widowed [ ]
Total Number of Persons in the Household:		1 – 5 Persons	6 – 10 Persons	11 + Persons
Status of Respondent's Parents:	Father Alive	Father Deceased	Mother Alive	Mother Deceased
**If father is alive, he lives in:**	Same household	Same town/village but different household	Different town/village	
**If mother is alive, she lives in:**	Same household	Same town/village but different household	Different town/village	

B. FAMILY HISTORY OF VASCULAR RISK FACTORS:

History of stroke in father, mother or sibling:	No	Yes, Father	Yes, Mother	Yes, Sibling
History of MI in father, mother or sibling:	No	Yes, Father	Yes, Mother	Yes, Sibling
History of DM in father, mother or sibling:	No	Yes, Father	Yes, Mother	Yes, Sibling
History of HTN in father, mother or sibling:	No	Yes, Father	Yes, Mother	Yes, Sibling

C. PERSONAL HISTORY OF VASCULAR RISK FACTORS – (VERIFY FROM CASE NOTES, PLEASE):

Stroke or transient ischemic attack (TIA):	No	Yes, I had a stroke	Yes, I had a TIA
Myocardial infarction or angina:	No	Yes, I had an MI	Yes, I have/had angina
Pulmonary thromboembolism (PTE):	No	Yes, I had PTE	Yes, I have PTE
Hypertension or Heart failure (HF)	No	Yes, I have HTN	Yes, I have heart failure
Atrial fibrillation (AF) or other arrhythmias:	No	Yes, I have AF	Yes, I have other arrhythmia
Obesity or Dyslipidaemia (DYSL):	No	Yes, I've/had obesity	Yes, I have/had DYSL
Cigarette smoking or excess alcohol use:	No	Yes, I smoked/smoke	Yes, I take excess alcohol
Use of amphetamines or other illicit drugs:	No	Yes, amphetamines	Yes, other illicit drugs
Other risk factor (eg, SCD, berry aneurysm):	No	Yes (*specify*):	

D. DIABETES CARE & COMPLICATIONS – (VERIFY FROM CASE NOTES, PLEASE):

Date of diabetes diagnosis:	< 1 Month	1 Mo. – 1 Year	1 – 10 Years	> 10 Years	
Length of follow-up at Diabetes Clinic:	< 1 Month	1 Mo. – 1 Year	1 – 10 Years	> 10 Years	
Current anti-diabetic treatments: (✓*Tick each one that applies*):	Diet	Oral glucose-lowering drug	Oral GLD with Insulin	Insulin only	Non-insulin injectable
Current dietary treatment for co-morbidities:	Hypertension	Dyslipidemia	Hyperuricemia		
	PUD	Other (*Specify*):			
Current drug treatment for co-morbidities: (✓ *Tick one or more*):	Anti-hypertensives	Lipid-lowering therapy	Antibiotics		
	Anticoagulants	Antiplatelets	NSAIDS		
	A. Anti-hypertensive drug: 1. 2. 3. 4.				
	B. Lipid-lowering drug: 1. 2.				

	3.	4.
	C. Antibiotic drug:<br>1.	2.
	C. Anticoagulant drug:<br>1.	2.
	D. Antiplatelet drug:<br>1.	2.
	E. NSAID drug:<br>1.	2.
	F. Other drug:<br>1.<br>3.	2.<br>4.

DM complications EVER experienced by respondent beside TIA, stroke, angina , MI and PTE (*tick as appropriate*):	DKA or HHS	Hypoglycemia	Skin abscess	Cellulitis
	Pyelonephritis	Other UTI	Necrotizing fasciitis	
	Mucormycosis		Generalised sepsis	
	Diabetic foot/ hand syndrome		CKD/ DM Nephropathy	
	DM retinopathy/ Cataract		Peripheral neuropathy	
	Autonomic neuropathy		Other complication	

Other DM complication (*please specify diagnosis and date):*
1.
2.
3.
4.

E. RESPONDENT'S KNOWLEDGE OF VASCULAR RISK FACTORS (Interviewer should NOT provide clues):

Respondent mentioned **SOME** of the 12 risk factors below, **SPECIFICALLY**:	
Hypertension	Yes [ ]
Diabetes mellitus	Yes [ ]
Obesity	Yes [ ]
Elevated serum cholesterol/ Dyslipidemia	Yes [ ]
Cigarette smoking	Yes [ ]
Heart disease	Yes [ ]
Excess use of alcohol	Yes [ ]
Lack of exercise	Yes [ ]
Illicit drug use	Yes [ ]
Old age	Yes [ ]
Family history of stroke	Yes [ ]
Family history of heart attack	Yes [ ]
Total Score:	

F. RESPONDENT'S KNOWLEDGE AND ATTITUDE TOWARDS EXERCISE:

*1. Does regular exercise help to lower blood glucose? **(If "NO", jump to "G" below)***	Yes	No	Don't know
2. Is 90 minutes of exercise per week adequate for people with diabetes?	Yes	No	Don't know
3. Can 2 hours of cooking or washing clothes substitute for a 1-hour walk?	Yes	No	Don't know
4. Could a diabetes patient continue with exercise when feeling chest pain?	Yes	No	Don't know
5. Should someone with diabetes rest for 3 days after vigorous exercise?	Yes	No	Don't know

G. RESPONDENT'S EXERCISE HABITS:

1. Do you exercise in at least three days every week?	No	Yes
2. Do you exercise for a total of 150 minutes or more every week?	No	Yes

	3. What form of exercise is your habit?	***Walking***	***Jogging***	***Push-up***	***Other (specify):***
	4. If you don't exercise, what is the reason?	*I didn't know that exercise is useful*			
		I knew exercise is useful but I just don't like it			
		I wanted to exercise but something prevented me			
	5. If you tried but failed to exercise, what had prevented you?	***A. Busy schedules at my job/office***			
		B. Childcare/pregnancy/household chores			
		C. Joint pain/body pain/breathlessness/imbalance of gait			
		D. Lack of exercise space in my household			
		E. Lack of a gymnasium/sports facilities in my area			
		F. Fear of accident/injury/crime in my area			
		H. My religion/culture forbids exercise outside my home			
		I. Other reason (specify):			

H. CLINICAL / LABORATORY FINDINGS:

	Height:	Weight:	Waist Circ (cm):	FBS:	HbA1C:	Urea:	Crea:
	GFR:		Total Chol:	LDL Chol:	HDL Chol:		Triglyc:
	Urinalysis:		Protein: (Yes) (No)	WBC:	Nitrites:		Blood:

Renal Ultrasound Scan Findings:
1.
2.
3.
4.
5.
6.

**I.**	**INTERVIEWER 1 (FIRST FOLLOW-UP)**<br>**NAME:**	**SIGNATURE:**	**DATE:**
	**INTERVIEWER 2 (SECOND FOLLOW-UP)**<br>**NAME:**	**SIGNATURE:**	**DATE:**
